# Establishment of a stable, effective and universal genetic transformation technique in the diverse species of *Brassica oleracea*

**DOI:** 10.3389/fpls.2022.1021669

**Published:** 2022-10-12

**Authors:** Xiaoguang Sheng, Huifang Yu, Jiansheng Wang, Yusen Shen, Honghui Gu

**Affiliations:** Institute of Vegetables, Zhejiang Academy of Agricultural Sciences, Hangzhou, China

**Keywords:** *Brassica oleracea*, cauliflower, broccoli, curd peduncle explants, direct shoot regeneration, effective transformation

## Abstract

*Brassica oleracea* is an economically important species, including seven cultivated variants. *Agrobacterium*-mediated transformation of *B. oleracea* crops, mainly *via* hypocotyl and cotyledon, has been achieved in the past. However, previously established transformation methods showed low efficiency, severe genotype limitation and a prolonged period for transformants acquisition, greatly restricting its application in functional genomic studies and crop improvement. In this study, we have compared the shoot regeneration and genetic transformation efficiency of hypocotyl, cotyledon petiole and curd peduncle explants from twelve genotypes of cauliflower and broccoli. Finally, an *Agrobacterium*-mediated transformation method using curd peduncle as explant was established, which is rapid, efficient, and amenable to high-throughput transformation and genome editing. The average genetic transformation efficiency of this method is stable up to 11.87% and was successfully implemented in twelve different genotypes of cauliflower and broccoli and other *B. oleracea* crops with low genotype dependence. Peduncle explants were found to contain abundant cambial cells with a strong cell division and shoot regeneration ability, which might be why this method achieved stable and high genetic transformation efficiency with almost no genotype dependence.

## 1 Introduction

*Brassica oleracea* is an economically important and outcrossing species domesticated from wild cabbage as early as 2000 BCE and has been classified into many unique morphotypes, including cabbage (var. *capitata*), kale (var. *acephala*), brussels sprout (var. *gemmifera*), Chinese kale (var. *albiflora*), kohlrabi (var. *caulorapa*), broccoli (var. *italica*) and cauliflower (var. *botrytis*) (Smyth, 1995). The crops of *B. oleracea* are widely grown in China, India, Italy, and other countries located mainly in Asia and Europe (FAO, 2018), which are rich in nutrients including antioxidants, vitamin A, vitamin K, β-carotene, and sulforaphane glucosinolate with anti-cancer properties (Ahmed and Ali, 2013; Chaudhary et al., 2014).

The early domestication of *B. oleracea* crops is generally assumed to take place in the Mediterranean and, eventually spread to different countries of Asia, Europe and America through trade (Snogerup, 1980; Branca et al., 2012). Previous studies have shown that *B. oleracea* species have a low genetic diversity indicating a narrow genetic base (Branca et al., 2012; Rakshita et al., 2021). Environmental stresses, diseases and insects cause substantial yield and quality losses to *B. oleracea* crops. Genetic engineering, including targeted genome editing technologies, could overcome sexual incompatibility barriers and improve the efficiency of conventional breeding, so it is becoming one of the most important approaches to improve their resistance to biotic and abiotic stresses. Moreover, *de novo* genome sequencing and chromosome assembly of *B. oleracea* crops were successfully completed recently, which would facilitate the cloning of target genes controlling beneficial traits (Belser et al., 2018; Sun et al., 2019; Guo et al., 2021). However, the precondition for delivering genes and genome editing is the accessibility of efficient transformation systems.

*Agrobacterium*-mediated transformation method is popular for transferring genes within *B. oleracea* crops because of the high proportion of single copy and stable transformation efficiency (Ravanfar et al., 2017). The shoot regeneration studies in *B. oleracea* crops have been widely reported, mostly using hypocotyls, cotyledon petioles and true leaf blocks as explants (Bhalla and Smith, 1998; Ravanfar et al., 2009; Farzinebrahimi et al., 2012; Kumar et al., 2015a; Kumar et al., 2015b; Kumar and Srivastava, 2016). The *Agrobacterium*-mediated transformation has been achieved firstly with several reporter or marker genes (*npt-II*, *gus* and *gfp*) (Christey et al., 1997; Cogan et al., 2001; Bhalla and Singh, 2008; Dhiman et al., 2014; Ravanfar and Aziz, 2014), followed by some functional genes including flowering control by *SLG*, *FCA*, *CONSTANS*, insect resistance by *cryIA*(*B*), *cry1A*(*C*), and delayed post-harvest yellowing or prolonged shelf-life by *SAD*, *ACC* oxidase 1, *BoCLH1* (Toriyama et al., 1991; Metz et al., 1995; Cao et al., 1999; Gapper et al., 2002; Viswakarma et al., 2004; Chen et al., 2008). However, in most of the published articles, the transformation has been met with limited success. Several drawbacks remain to be solved, such as low transformation efficiency, high dependence on genotype and a prolonged period of calli induction delaying resistant shoots regeneration, which hinders further development of this technology and also prevents its application in gene editing (Kumar and Srivastava, 2016).

The purpose of this study was to establish an efficient and universal *Agrobacterium tumefaciens*-mediated transformation system in *B. oleracea* crops, especially in cauliflower and broccoli. We evaluated the shoot regeneration efficiency in different explants, including hypocotyl, cotyledon petiole and curd peduncle from twelve different genotypic lines. Subsequently, we investigated the factors affecting *Agrobacterium*-mediated transformation efficiency, such as genotypes, explant types, antibiotics concentration and infection duration. The optimized peduncle-based system produced a stable transformation efficiency with an average of 11.87% with almost no genotype dependence. The regenerated transgenic plants still retained the memory of passing vernalization and could flower without an additional process of vernalization, which greatly accelerated the acquisition of homozygous transgenic plants of the T_1_ generation. Moreover, this system was also successfully applied to other *B. oleracea* crops, including cabbage and Chinese kale. Overall, the system developed in the present study can be applied in functional genomics research and traits improvement of cauliflower and broccoli, which can also be extended to other *B. oleracea* crops, such as Chinese kale and cabbage.

## 2 Materials and methods

### 2.1 Plant materials

Twelve pure lines of cauliflower and broccoli, including DH lines (developed through microspore culture) and inbred lines (F_6-8_) (**Table S1**) were used in the present study. The seeds of these pure lines were collected from the Institute of Vegetables of Zhejiang Academy of Agricultural Sciences, Hangzhou, Zhejiang Province, which has been engaged in the breeding of cauliflower and broccoli for more than fifteen years. For *in vitro* germination, seeds were surface-sterilized in 70% ethanol for 40 s and then immersed in 3% sodium hypochlorite for 10 min, followed by rinsing 3-4 times with sterile ddH_2_O. The seeds were then placed on the MS medium added with 20 g sucrose and 9 g agarose in one liter of solution (pH 5.8-6.0) and incubated at 22/20°C (day/night), a light cycle of 14/10 h (day/night), and light intensity of approximately 100 μmol m^–2^ s^–1^. After six days of cultivation, the explants of hypocotyls and cotyledons with petioles were cut off and used for *Agrobacterium*-mediated transformation. For harvesting the peduncles explants, seeds were directly sown in a multicellular tray filled with substrate and then transplanted into the soil condition in a greenhouse at the seedling stage with 3-4 true leaves. When the flower head matured, peduncles were harvested and surface-sterilized, as mentioned above. The tested cabbage and Chinese kale materials are bought from the market, named Zhonggan 21 and J1401, respectively.

### 2.2 Overexpression vector construction and *Agrobacterium* strain

*Agrobacterium tumefaciens* strain GV3101 harboring plasmid of pCambia1301 was used in this study. The *BoTFL1* gene highly expressed in the curd development stage of cauliflower was selected to construct the target vector (Sheng et al., 2020). The coding region of *BoTFL1* was amplified using cDNA generated from the curd of cauliflower (with primers of KpnI-F and KpnI-R) and was then sub-cloned into the binary plasmid vector of pCambia1301 carrying hygromycin phosphotransferase (*HPT*), neomycin phosphotransferase II (*nptII*) and *GUS* gene (**Table S2**).

### 2.3 *Agrobacterium*-mediated plant transformation

Three types of explants including, hypocotyl, cotyledon petiole and peduncle, were pre-cultured for 5 d in MS1-1and MS1-2 medium, respectively, at the temperature of 22/20°C (day/night), a light cycle of 14/10 h (day/night), and light intensity of approximately 100 μmol m^–2^ s^–1^. Then, all the explants were infected by the plasmid-harboring *Agrobacterium* with a bacterial concentration of OD_600_ = 0.8-1.0 for different time duration. After 2 days of co-culture in the dark on MS1-1and 2 medium, all the explants were transferred to MS2-1 and 2 medium for 7 d of recovery culture, respectively. After that, all the explants were transferred to the selection medium of MS3-1 and 2 medium, respectively, and the mediums were renewed every fifteen days until the resistant shoots emerged. Then, the hygromycin-resistant plants were cut from the explants and transferred to an MS4 medium to induce root growth. The rooted plantlets were transplanted into a small nutrient bowl filled with substrate and cultured in an artificial climate chamber for two weeks with a temperature of 22/20°C (day/night), a light cycle of 14/10 h (day/night), and light intensity of approximately 100 μmol m^–2^ s^–1^. Then the plantlets were transplanted in the soil condition in a specially isolated greenhouse and continued to grow until the flowering and seed setting stage. The detailed components of all the above culture mediums are shown in **Table S3**.

### 2.4 Confirmation of transformants

#### 2.4.1 Polymerase chain reaction (PCR) analysis

Putative transformants were pre-screened by PCR. A common cetyltrimethylammonium bromide (CTAB) method was used to extract the genomic DNA of each hygromycin-resistant plant (Doyle, 1987). The constructed plasmid vector of pCambia1301 and untransformed wild-type plants were used as positive and negative controls, respectively. A specific primer set, forward 5’- CGATTGCGTCGCATCGACC -3’ and reverse 5’ - TTCTACAACCGGTCGCGGAG -3’, was selected to amplify a 0.58 kb fragment specific to the *HPT* gene. PCR was carried out in a total volume of 20 μL reaction mixture comprising 12.5 μL of the PCR mix (Takara, ExTaq), 1 µL of genomic DNA (50-100 ng/μL), 0.75 μL of each primer and 5 μL of double distilled sterile water. The PCR cycle conditions were as follows: 5 min, 94°C; 20 s, 94°C, 20 s, 58°C, and 30 s, 72°C for 30 cycles; and 10 min, 72°C. The amplified products of the *HPT* gene were electrophoresed in 1% agarose gels and viewed under a UV transilluminator.

### 2.5 The analysis of histochemical *GUS* staining

The PCR-positive plants were selected for *GUS* staining assay for further confirmation of the insertion of the target T-DNA into the recipient genome. The untransformed plants were selected as the negative control. At the stage of *in vitro* culture, one leaf from each plantlet was sampled and immersed in a 2-mM X-Gluc solution (Leagene, DP0013) overnight at 30°C. At the curd maturity stage and blooming, the small branches, buds and flowers were cut and immersed in the same X-Gluc solution, which was vacuumed for 30 minutes (0.01 MPa), and then kept overnight at 30°C. After staining, the tissues were washed in 75% ethanol three to five times until no chlorophyll content was visible. The explants with at least one blue spot were determined to possess the transient *GUS* expression.

### 2.6 Cryo-scanning electron microscope (cryo-SEM) observation

To further ascertain the microstructure of the explant transverse incision sites clearly, a cryo-SEM was applied (HITACHI S-3000N&Quorum PP3000T, Japan and England) according to the procedures presented by Kotula et al. (2019). After 30 minutes of *Agrobacterium* infection and one day of co-cultivation, the interface of explants was cross-sectioned and cryo-fixed in a carbon film copper grid with liquid nitrogen. The carrier grid with samples was then quickly delivered to a cryo-preparation chamber under vacuum. After that, the frozen samples were sublimated for 6 min at − 80°C and then delivered to an SEM chamber and conducted for microstructure observations.

### 2.7 Histology observation

The fresh explants were cut into approximately 1 cm long segments and then fixed in Farmer’s Fixative (3:1 ethanol: acetic acid) for 24 h at 4°C. The subsequent procedures of dehydration, embedment and section were conducted as described by Oi et al. (2022). Images were taken under a D72 light microscope (Olympus). Three biological replicates were performed for each explant.

### 2.8 Data analysis

Factors of the genotypes and explants were evaluated in this study. At least three biological replicates for each factor were analyzed, and each biological replicate consisted of 50-60 explants for shoot regeneration and 80-90 explants for *Agrobacterium*-mediated transformation. The SPSS software of version 16.0 was selected for statistical analysis of all the experimental data in this study, and the significant differences among treatments were set at P < 0.05.

## 3 Results

### 3.1 Effect of genotype and explant on shoot regeneration efficiency

**Figure 1:**
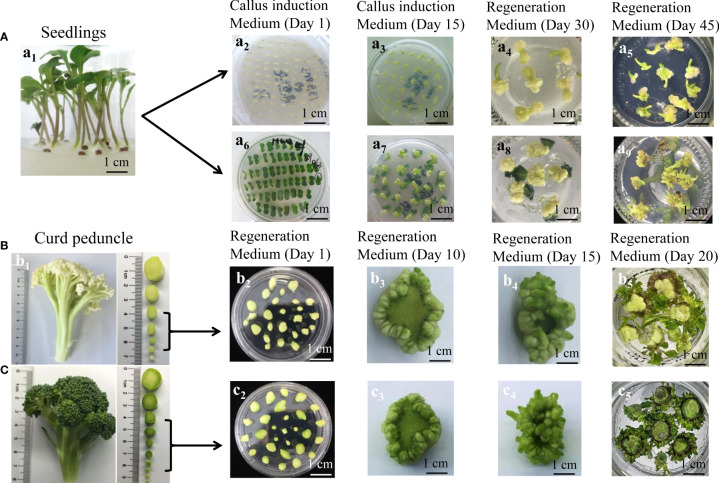
The shoot regeneration from different explants of C90IL-9 and B80IL-2. **(A)** shoot regeneration from hypocotyl and cotyledon petiole explants of C90IL-9. (A1), seedlings grown on sterile medium for 6 d after seed sterilization, Calli induction and shoot regeneration from hypocotyl (A2-5) and cotyledon petiole (A6-9) from day 1 to day 45. **(B)** and **(C)** shoots directly regenerated from curd peduncle explants. A branch from a mature curd of C90IL-9 (B1) and B80IL-2 (C1), respectively, and the peduncles with cross-sectional diameter of 0.3-0.8 cm were selected for shoot induction culture from day 1 to day 20 [(B2-5) of C90IL-9 and (C2-5) of B80IL-2].

Two accessions (C90IL-9 of cauliflower and B80IL-2 of broccoli) were selected to evaluate the shoot regeneration efficiency of three types of explant, including hypocotyl, cotyledon petiole and curd peduncle. Hypocotyl and cotyledon petiole were excised from seedlings grown on a sterile medium for 6 d after seed sterilization (**Figure 1A**). The curd peduncle was taken from the mature curd of cauliflower and broccoli. After sterilization, the curd peduncles were cut into small segments of about 0.5 cm long and placed on the shoot induction medium. According to the diameter of the peduncles cross-section, they were divided into four grades; 0.1-0.2 cm (grade 1), 0.3-0.5 cm (grade 2), 0.6-0.8 cm (grade 3) and 0.9-1.3 cm (grade 4) (**Figures 1B, C**). The hypocotyls and cotyledon petioles were cultivated on callus induction medium for 21 d and then cultured in regeneration medium for another 30-40 d until successful shoot regeneration (**Figure 1A**). For the accession of C90IL-9, the shoot regeneration efficiencies of the hypocotyl and cotyledon petiole explants were 85.13% and 80.69%, and they developed 3.52 and 5.35 shoots per explant, respectively. The shoot regeneration efficiencies of both explants from the accession of B80IL-2 were 90.16% and 80.52%, respectively, producing 3.82 and 6.17 shoots per explant (**Table 1**). The curd peduncle explants of both accessions cultured on a bud induction medium for 25-30 days can directly regenerate buds without callus induction (**Figures 1B, C**). The shoot regeneration efficiency of curd peduncles at grade 2 and grade 3 were higher, which could achieve more than 94% and produce 7.79 to 11.21 shoots per explant.

**Table 1:** Effect of explants on shoot regeneration rate of C90IL-9 and B80IL-2.

Source	Variety	Type	No.	Regeneration efficiency (%)	Number of shoots per explant
Curds	C90IL-9	Peduncles at grade 1	148	85.13 ± 3.32 ab	3.69 ± 0.82 ab
		Peduncles at grade 2	156	94.45 ± 1.76 d	7.79 ± 1.74 cd
		Peduncles at grade 3	159	95.64 ± 1.23 d	10.38 ± 2.02 de
		Peduncles at grade 4	152	82.37 ± 3.68 a	5.43 ± 1.64 abc
	B80IL-2	Peduncles at grade 1	142	87.65 ± 2.31 bc	2.74 ± 0.66 a
		Peduncles at grade 2	161	95.81 ± 2.06 d	8.32 ± 2.83cde
		Peduncles at grade 3	154	97.43 ± 1.31 d	11.21 ± 2.49 e
		Peduncles at grade 4	151	84.17 ± 2.68 ab	4.21 ± 1.47 ab
Seedlings	C90IL-9	Hypocotyls	269	85.13 ± 1.39 ab	3.52 ± 0.83 ab
		Cotyledon petioles	254	80.69 ± 3.54 a	5.35 ± 1.46 abc
	B80IL-2	Hypocotyls	257	90.11 ± 1.97 c	3.82 ± 0.92 ab
		Cotyledon petioles	248	82.72 ± 3.08 a	6.17 ± 1.23 bc

Different letters (a-e) after the levels of each factor indicate the statistically significant differences (P < 0.05).

**Figure 2:**
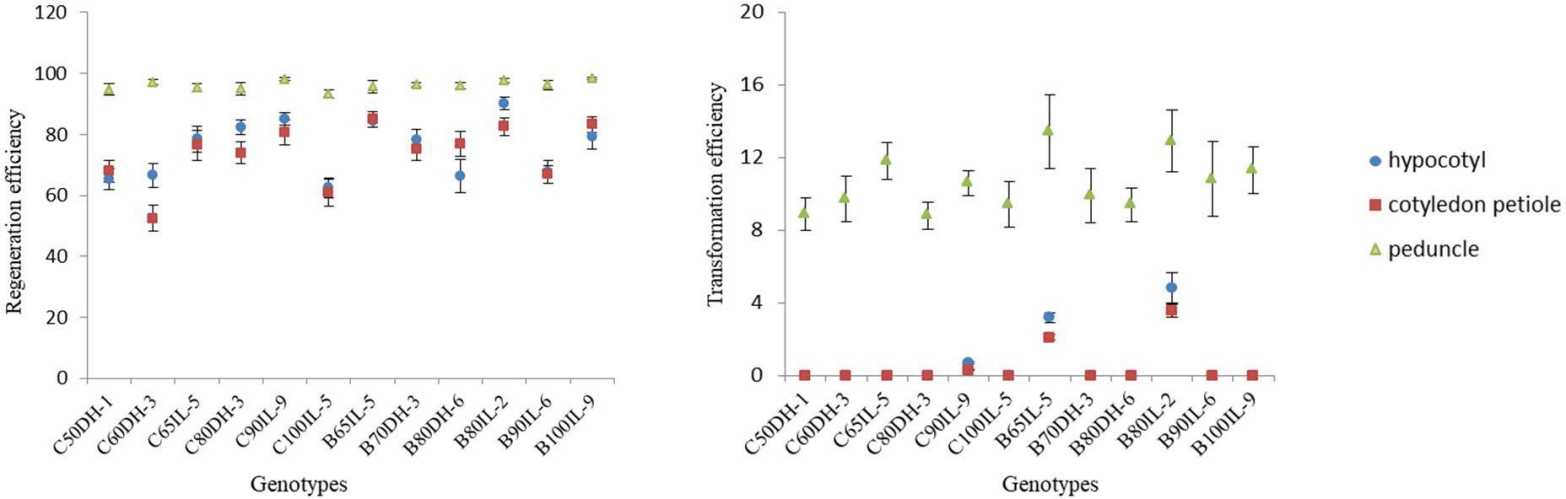
Effect of genotypes on the shoot regeneration and transformation efficiency of different explants. The regeneration and transformation rate were calculated as the percentage of the explants regenerated shoots and positive regenerated plants/total explants, respectively, for each genotype.

Genotype dependence is the main limiting factor for successful shoot regeneration and *Agrobacterium*-mediated transformation in *Brassica* crops. In order to improve the universality of this protocol, in the present investigation, we have carried out the regeneration assays for twelve core breeding lines of broccoli and cauliflower with different ecotypes and genotypes. For the explants of hypocotyl and cotyledon petiole, significant differences were observed between the accessions. B80IL-2 attained the highest shoot regeneration efficiency of 90.09%, and C60DH-3 showed the lowest of 52.43%. Peduncle explants exhibited overall high regeneration efficiency ranging from 93.26% to 98.34%, with an average of 96.29%, with no significant differences among the accessions (**Figure 2**, **Table S4**). In general, achieving more than 50% regeneration efficiency is regarded as efficient in genetic transformation experiments. Therefore, the next step of *Agrobacterium*-mediated genetic transformation was carried out for all the genotypes and different explants so as to select the optimal combination of explant and genotype.

### 3.2 Effect of explant, genotype, and Agrobacterium-immersion parameters on transformation efficiency

For *Agrobacterium*-mediated transformation, we selected the over-expression vector pCMABIA-1301 with the homologous gene of *TERMINAL FLOWER 1* in cauliflower and broccoli (*BoTFL1*) and marker gene *GUS*, which are under the control of CaMV35S promoter. Three types of explants, including hypocotyl, cotyledon petiole and curd peduncle from the above twelve genotypes of cauliflower and broccoli, were used for *Agrobacterium*-mediated transformation. The tolerance of peduncle explants to *Agrobacterium* tumefaciens was significantly higher than that of hypocotyl and cotyledon. After 10 minutes of *Agrobacterium* infection, the browning rate of hypocotyl and cotyledon increased significantly and exceeded 46% after 20 minutes of immersion. The peduncle explants could withstand the infection time of 30 minutes, and then the browning rate increased with the extension of infection time. After 60 minutes of infection, the browning rate of the curd peduncle reached about 40% (**Table S5**). Therefore, the infection time of 10 minutes for hypocotyls and cotyledons and 30 minutes for peduncle were considered to be most suitable for all the genotypes. The regenerated buds from various explants of cauliflower and broccoli were sensitive to hygromycin, which at a concentration of 10 mg/L could inhibit more than 80% of the regeneration efficiency. The results of three independent genetic transformation experiments showed that over 60% of the survived plantlets were positive for both PCR and *GUS* activity under the selection of 10 mg/l hygromycin.

According to the genetic transformation process shown in **Figure S1**, the transgene-positive plants were obtained only from the hypocotyl and cotyledon petiole explants of the three genotypes, and the transformation efficiency was as low as 0.3-4.8%. Conversely, transgenics were achieved from peduncle explants of all the genotypes, and the average transformation efficiency ranged from 8.8 to 13.4%. Additionally, *Agrobacterium*-mediated transformation of hypocotyl and cotyledon petiole explants took a longer time, about 70-80 days, to obtain transgene-positive plants because of the requirement of a long recovery time after *Agrobacterium* infection and an extended period of callus induction and growth. In contrast, the peduncle explants could successfully regenerate transgene-positive plants within 35-40 days because of their strong tolerance to *Agrobacterium tumefaciens*, rapid recovery of post-infection, and the ability to regenerate buds directly with almost no callus induction.

**Figure 3:**
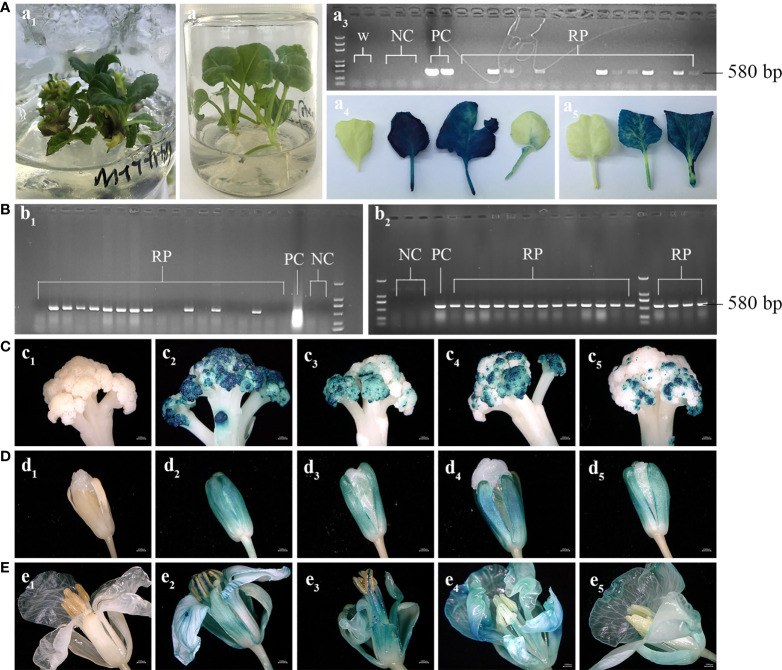
PCR analysis and histochemical *GUS* staining of transgenic plants at the stages of aseptic seedlings **(A)** and flowering after transplanted into soil condition in greenhouse **(B–E)**. (A1) hygromycin resistant shoots, (A2) hygromycin resistant plants regenerated adventitious roots, a3, PCR analysis of transgenic plants using a pair of specific primers for *HPT* gene and a target band of 580 bp was amplified from the positive plants. Histochemical *GUS* staining for the leaves of transgenic seedlings and those with blue spots were positive plants (A4, A5). (B1) and (B2), the positive plants of cauliflower (B1) and broccoli (B2) identified at seedling stage were transplanted into the soil for the second round of PCR analysis. The second round of histochemical *GUS* staining analysis of cauliflower curd (C1, negative control, (C2-5) transgenic plants) and broccoli flowers (D1 and C1, negative control, D2-5 and E2-5, transgenic plants). w, water. NC, negative control. PC, positive control. RP, regenerated hygromycin resistant plants.

A total of 38 and 56 lines of broccoli and cauliflower, respectively, with positive PCR and *GUS* activity, were transplanted in the soil condition (**Figure 3A**). During blooming, the second round of PCR and *GUS* activity detection tests were carried out to verify the authenticity of the transgenic plants. Interestingly, eight cauliflower plants lost the target band in PCR detection, whose leaves and buds also showed no *GUS* activity, indicating that they lost the target T-DNA during growth. The other 48 cauliflower plants preserved the target T-DNA insertion based on PCR and *GUS* staining results (**Figures 3B-b1, C**). However, all broccoli plants amplified the target band in the second round of PCR detection, and the sepals, petals and anthers showed strong *GUS* activity, confirming stable T-DNA insertion within the broccoli genome (**Figures 3B-b2, D, E**).

### 3.3 High tolerance of peduncle explants to Agrobacterium improves its transformation efficiency

**Figure 4:**
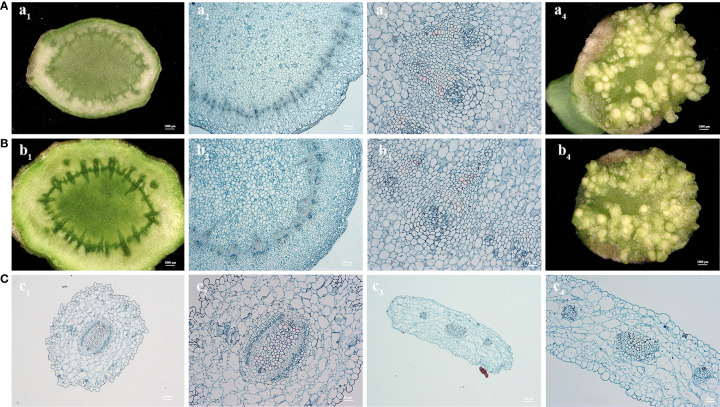
Cross section observation of paraffin embedded tissues from explants of curd peduncle **(A, B)**, hypocotyl and cotyledon petiole **(C)**. Cross section of fresh peduncle explants of C90IL-9 (A1-3) and B80IL-2 (B1-3). Shoot induction of peduncle of C90IL-9 (A4) and B80IL-2 (B4) after 10 days of culture in shoot induction medium. Cross section of fresh hypocotyl (C1-2) and cotyledon petiole (C3-4) explants of C90IL-9.

Peduncle explants are similar to young stems and have a nearly cylindrical shape. The section of the peduncle explant is nearly circular, and the circumferential layer near the epidermis contains a large number of meristem cells, which possess extremely strong meristematic ability (**Figures 4A, B**). Under hormonal stimulation, shoot emergence from the meristem cells can be seen at the incision site with the naked eye after 10 days of culture, and the whole plant can be regenerated after 25-30 days. After *Agrobacterium tumefaciens* infection, cambial cells were observed to have a strong recovery ability. Shoot regeneration was restored approx. 10 days after infection, and the resistant plants were regenerated 35-40 days after infection. Conversely, the microtubule tissue of hypocotyl and cotyledon petiole was underdeveloped. From the cross-sectional pictures, a large area of loosely arranged epidermal cells with large vacuoles and few vascular bundles in the center was observed (**Figure 4C**). Therefore, the direct regeneration ability of hypocotyl and cotyledon petiole was found to be poor, which required a process of callus induction to regenerate shoots.

**Figure 5:**
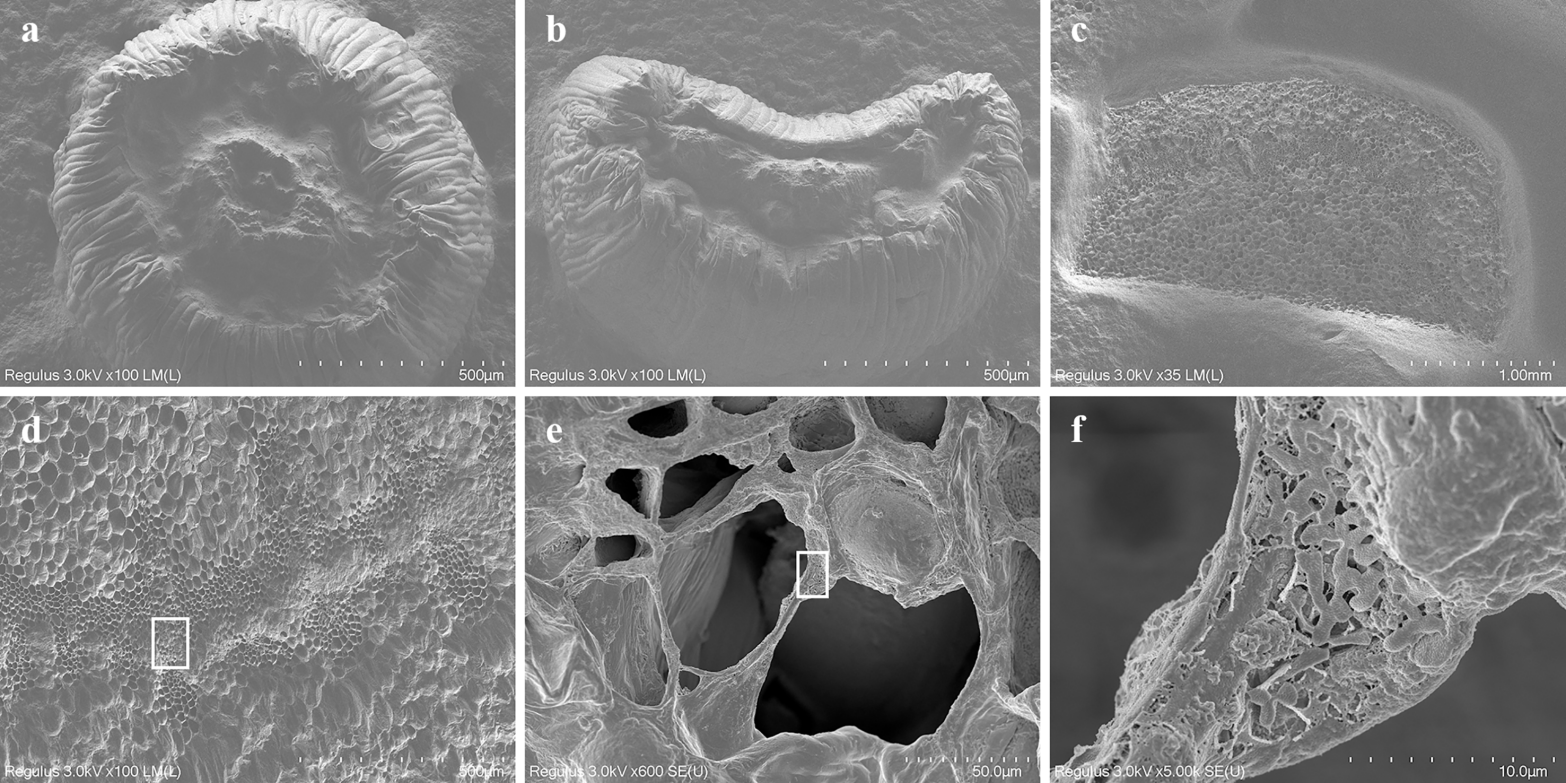
Cryo-electron microscopic observation of incisional tissues of three different explants. The incision morphology of hypocotyl **(A)**, cotyledon petiole **(B)** and curd peduncle **(C)** after infected with *Agrobacterium tumefaciens* and co-cultured for one day. **(D–F)** incision morphology of peduncle explants under different magnification and many *Agrobacterium tumefaciens* were found in the vascular bundle near the cambium cells **(F)**.

The cross-sectional diameter of the peduncle explant ranged from 0.3 to 0.8 cm, the area of which is much larger than that of the hypocotyl and cotyledon petiole (**Figure 1**). The results of freeze electron microscopy showed that the peduncle incision was normal after pre-culture, which was effective for full contact with *Agrobacterium tumefaciens*. After *Agrobacterium* infection and co-cultured for 1 day, a large number of *Agrobacterium tumefaciens* were found in the vascular bundle near the cambium cells of peduncle explants (**Figure 5**). This might have happened due to the release of chemotactic substances such as acetosyringone by peduncle explants inducing the accumulation of *Agrobacterium tumefaciens*, thereby increasing the transformation rate of incision cells. In contrast, the incisions of hypocotyl and cotyledon petiole were greatly shrunken after pre-culture, which largely reduced the contact area with *Agrobacterium*. In addition, no accumulation of *Agrobacterium* was found in the wounds of hypocotyl and cotyledon petiole explants (**Figure 5**).

### 3.4 Curd peduncle-based transgenic plants possess the ability to flower directly

**Figure 6:**
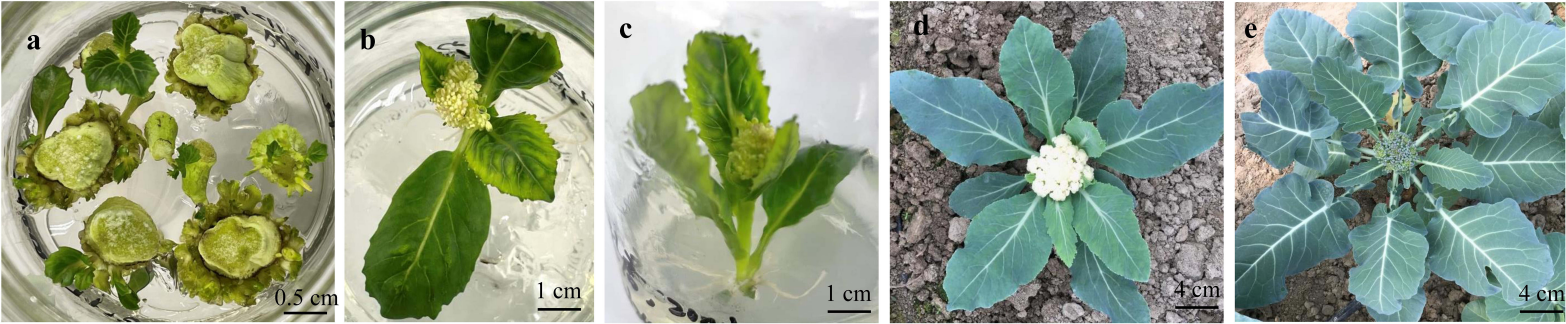
Transgenic plants regenerated from peduncle explants can directly grow curds and flower without going through low-temperature of vernalization. **(A)** resistant shoot screening of the peduncle explants from C60DH-3. **(B, C)** the regenerated plants of B65IL-5 **(B)** and C60DH-3 **(C)** budded in the tissue culture bottles. **(D)** transgenic plants of C90IL-9 were transplanted into soil, and the curd began to appear after 8-9 true leaves were grown. **(E)** transgenic plants of B100IL-9 were transplanted into soil and budded after growing 9-10 true leaves.

*B. oleracea* crops go through a typical type of vernalization process, in which the plants need to grow to the mature vegetative stage and then undergo a period of low-temperature treatment to promote flowering. Peduncle explants derived from the curds in the reproductive growth phase could remember their prior vernalization and then pass it on through mitosis. Therefore, the transgenic plants regenerated from the explants of the curd peduncle do not need to undergo the process of vernalization and can bloom as long as there are proper illumination and temperature. g acceFor the late maturinssions, the plants regenerated from peduncle explants started showing curds (cauliflower) and buds (broccoli) at the 9-10 leaf stage, and the flowering time was significantly earlier in these plants. Moreover, the regenerated plants of some early and medium maturing genotypes bloom directly in sterile culture bottles (**Figure 6**).

### 3.5 Successful application of genetic transformation using peduncle explant in cabbage and Chinese kale

**Figure 7:**
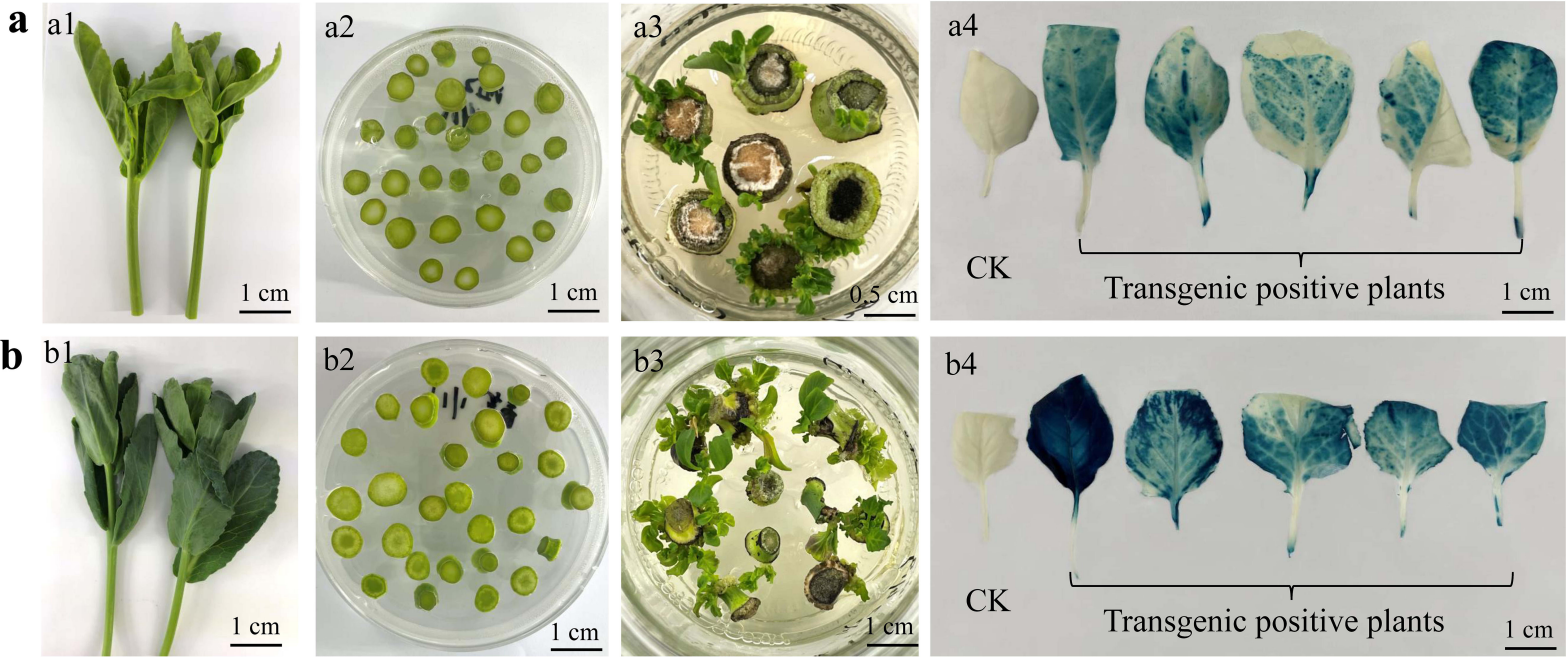
Transgenic plants regenerated from tender stems of lateral branches of Chinese kale **(A)** and cabbage **(B)**. The tender stems of lateral branches (A1, B1), which were cut into segments with a diameter of 0.3-0.8 cm (A2, B2). Resistant buds were regenerated from the tender stem explants (A3, B3) and the histochemical *GUS* staining of the leaves from transgenic positive plants (A4, B4).

The *Agrobacterium*-mediated genetic transformation technique based on peduncle explants established in this study was further tested in cabbage and Chinese kale to determine the universality of this method in other *B. oleracea* vegetable crops. The young budding lateral branches were selected and excised into different segments with a diameter of 0.3-0.8 cm. The sterilization and transformation methods followed the procedures presented in **Figure S1**. Resistant buds were regenerated from the tender stem explants of cabbage approx. 30 days after *Agrobacterium* infection. After the PCR amplification using primers specific to *HPT* gene and *GUS* histochemical staining of the regenerated plants, fifteen transgene-positive plants were obtained from 147 tender stem explants, and the average genetic transformation efficiency was 10.21%. The degree of lignification of the tender stem of Chinese kale was higher, and the browning rate after *Agrobacterium* infection was a little more severe compared with those of cabbage. However, the regeneration of transgenic-positive plants was also successfully obtained by this method, and the genetic transformation efficiency was about 6.82% (**Figure 7**).

## 4 Discussion

The tissue culture-based transformation technique has been widely used in gene editing and transgenesis to promote plant genomics studies. Although *Brassica* vegetables respond well to tissue culture and several published reports have demonstrated the successful regeneration of transformed plants in broccoli and cauliflower, the effectiveness of transformation is significantly correlated with the genotypes, and only a few genotypes have been reported to be successfully transformed (Chakrabarty et al., 2002; Chen et al., 2008; Ravanfar and Aziz, 2014). Moreover, many factors such as explant type, *Agrobacterium* concentration and immersion time also greatly influence the regeneration and transformation efficiency (Sharma and Srivastava, 2014; Kumar and Srivastava, 2016). Therefore, the establishment of an efficient, stable and general transformation system independent of genotype has always been one of the key points of genetic research in *Brassica* crops.

Hypocotyl and cotyledon petiole derived from sterile seedlings were the two most commonly used explants in genetic transformation research of *B. oleracea* crops, and some successful transformation events using the two types of explant have also been presented in several reports (Chakrabarty et al., 2002; Chen et al., 2008). However, these two types of explants-based genetic transformation systems showed strong genotype dependence, and the reproducibility of the experiments was not high (Chen et al., 2008). In general, genotypes with lower genetic transformation efficiency exhibited poorer experimental repeatability. The results of this study were in agreement with such an observation. Among the twelve genotypes, only three accessions regenerated transgene-positive plants with the transformation efficiency ranging from 0.3 to 4.8%. Additionally, hypocotyl and cotyledon petiole explants also require a process of dedifferentiation to form calli, which are then redifferentiated and eventually regenerate shoots (Wagoner et al., 1992; Suri et al., 2005). Dedifferentiation and redifferentiation phages of plant tissue culture is a long process, which greatly prolongs the time to regenerate positive plants and increases the frequency of potential somatic variants (Metz et al., 1995; Chen et al., 2001).

The peduncle explant used in the present study is a fleshy stem located below the inflorescence meristem and a small flower bud in the mature curd of cauliflower and broccoli. Timothy et al. (1995) and Chen et al. (2001) reported the successful regeneration of transformants using peduncle explant in broccoli, which exhibited higher genetic transformation efficiency than the hypocotyl explant. The findings of our study also confirmed the above observation. The peduncle explants from twelve genotypes of cauliflower and broccoli obtained up to 95% shoot regeneration efficiency, and all the accessions successfully regenerated transgenic plants with transformation efficiency ranging from 8.8 to 13.4%. Moreover, the peduncle-based genetic transformation technique established in this study is stable and can be applied to different genotypes of *B. oleracea* crops.

Peduncle explants, similar to young stems, contain typical tissues of the xylem, phloem and middle cambium. The cambium cells possess vigorous cell division and shoot regeneration ability (Liu et al., 2014; Alaguero-Cordovilla et al., 2021). In this study, we observed that many shoots have regenerated from the cambium cells of peduncle explants by direct organogenesis with no or few calli induction during the processes of *Agrobacterium* elimination and hygromycin selection. Due to the elimination of the callus induction process, the time required for shoot regeneration from peduncle explants was greatly shortened, and the vitrification rate and potential genomic variation rate of the regenerated shoots were lower than those of hypocotyl and cotyledon petiole explants (Chen et al., 2001). Moreover, peduncle explants express strong tolerance to *Agrobacterium tumefaciens*, which might be due to the support of developed vascular tissue, and the extension of *Agrobacterium*-infection time could improve the genetic transformation efficiency to a certain extent. Therefore, the developed cambium cells and microtubule tissue might be the reason for achieving high genetic transformation efficiency. These tissue-specific organ characteristics are less affected by genotype. Therefore, the peduncle explant-based transformation system established in this study could be applied to different genotypes of *B. oleracea* crops, including cauliflower and broccoli.

The species of *B. oleracea* includes seven cultivated variants. Since 2014, the whole genome sequences of cabbage (var. *capitata*) (Liu et al., 2014), kale (var. *acephala*) (Parkin et al., 2014), broccoli (var. *italica*) (Belser et al., 2018) and cauliflower (var. *botrytis*) (Sun et al., 2019; Guo et al., 2021), etc, have been published in succession, providing important references for the genetic localization and cloning of the target genes controlling important agronomic traits. Since then, the research on important gene mining and their regulatory mechanism of *B. oleracea* crops has entered a period of rapid development. A series of genetic loci or genes controlling plant growth, resistance to biotic and abiotic stresses, quality composition and fertility, etc., have been excavated (Li et al., 2021; Shaw et al., 2021; Zhang et al., 2021; Han et al., 2021; Ren et al., 2022; Alemán et al., 2022). Therefore, the effective transformation method established in this study will greatly accelerate the functional validation and regulatory mechanism study of the target genes controlling important agronomic traits of *B. oleracea* crops and promote rapid gene pyramiding to develop excellent target materials.

In conclusion, we have developed a stable, effective and genotype-independent *Agrobacterium*-mediated transformation system with the advantages of resistant shoots acquirement within a short period of 35-40 days, which could be successfully applied to different genotypes of broccoli and cauliflower, and even other species of *B*. *oleracea*. The established method has laid the foundation for further gene function studies through genetic transformation and mutant creation.

## Data availability statement

The original contributions presented in the study are included in the article/**Supplementary Material**. Further inquiries can be directed to the corresponding author.

## Author contributions

Conceptualization, HG and XS; methodology, XS and HY; software, YS; writing—original draft preparation, XS; writing—review and editing, XS, JW, and HY; supervision, HG; project administration, HG and JW. All authors have read and agreed to the published version of the manuscript.

## Funding

This work was funded by the Science and Technology Department of Zhejiang Province for Key Agriculture Development (2021C02065-4-1, 2021C02065-4-4), the Key R&D Program in Zhejiang Province (2021C02042, 2022C02051), Natural Science Foundation of Zhejiang Province (LD22C150002) and Project of Zhejiang Academy of Agricultural Sciences.

## Acknowledgments

We appreciate Dr. Sun Bo (College of Horticulture, Sichuan Agricultural University, China) for the guidance and help in the process of experiment.

## Conflict of interest

The authors declare that the research was conducted in the absence of any commercial or financial relationships that could be construed as a potential conflict of interest.

## Publisher’s note

All claims expressed in this article are solely those of the authors and do not necessarily represent those of their affiliated organizations, or those of the publisher, the editors and the reviewers. Any product that may be evaluated in this article, or claim that may be made by its manufacturer, is not guaranteed or endorsed by the publisher.
