# Supplementary material for: Establishment of a stable, effective and universal genetic transformation technique in the diverse species of Brassica oleracea
Source: Front Plant Sci. 2022 Oct 12;13:1021669. doi: 10.3389/fpls.2022.1021669 (PMC9597678; doi:10.3389/fpls.2022.1021669)
Supplement: Supplementary file 1 [file DataSheet_1.docx]

Supplementary Material

Table S1 Information of twelve lines for the test in this paper

| No. | Name | Species | Types | Source (varieties) |
| --- | --- | --- | --- | --- |
| 1 | C50DH-1 | cauliflower | DH line | Qingnong 45 |
| 2 | C60DH-3 | cauliflower | DH line | Qingnong 65 |
| 3 | C65IL-5 | cauliflower | >F_6_ | Xuebai 65 |
| 4 | C80DH-3 | cauliflower | DH line | Taisong 80 |
| 5 | C90IL-9 | cauliflower | >F_7_ | Qingnong 90 |
| 6 | C100IL-5 | cauliflower | >F_6_ | PLANITA |
| 7 | B65IL-5 | broccoli | >F_6_ | Sanxiong 138 |
| 8 | B70DH-3 | broccoli | DH line | Sano No.2 |
| 9 | B80DH-6 | broccoli | DH line | Sano No.99 |
| 10 | B80IL-2 | broccoli | >F_8_ | Sanxiong 56 |
| 11 | B90IL-6 | broccoli | >F_7_ | Lvxiong 90 |
| 12 | B100IL-9 | broccoli | >F_7_ | Sanxiong120 |

Table S2 The primers and target gene used in this study.

| Primer and gene name | Sequence (5’-3’) | Aims |
| --- | --- | --- |
| *Hyg*-F | CGATTGCGTCGCATCGACC | Detection of the hygromycin resistance gene |
| *Hyg*-R | TTCTACAACCGGTCGCGGAG |  |
| *KpnI*-F | GCGGGTCGACGGTACCATGGGAGAAAGAGTGATAGAG | Amplification of the full length of *BoTFL1* coding region carrying 16 base sequences at both ends of the *KpnI* single enzyme digestion site of pCAMBIA1301 vector |
| *KpnI*-R | TAGACATATGGGTACCTTAACGTCTTCGAGCTGCAG |  |
| *BoTFL1* | ATGGGAGAAAGAGTGATAGAGCCATTGATAATGGGAAGAGTGGTAGGAGATGTTCTCGATTTCTTCACTCCAACAATTAAAATGAATGTGAGCTACAACATGAAGCAAGTCTCCAACAGCCATGAGCTTTTTCCTTCCTCTGTCTCCTCCAAGCCTAGGGTTGAGATCCATGGTGGTGATCTCAGATCCTTCTTCACCTTGGTGATGATAGACCCTGATGTTCCAGGTCCTAGTGACCCCTTTCTAAAAGAGCACCTGCATTGGATAGTAACAAACATCCCCGGTACAACCGATGCTACATTTGGAAAAGAGGTGGTGAGCTATGAGTTGCCAAGGCCTAGCATAGGGATACACAGGTTCGTGTTTGTTCTGTTCAAGCAGAAGCAAAGACGTGTTATCTTCCCAAACATTCCTTCGAGAGATAACTTCAACACTCGAAAATTTGCGATCGAGTATGATCTTGGTCTTCCTGTCGCTGCTGTCTTCTTTAACGCCCAGAGAGAAACTGCAGCTCGAAGACGTTAA | The target gene of pCAMBIA1301 overexpression vector  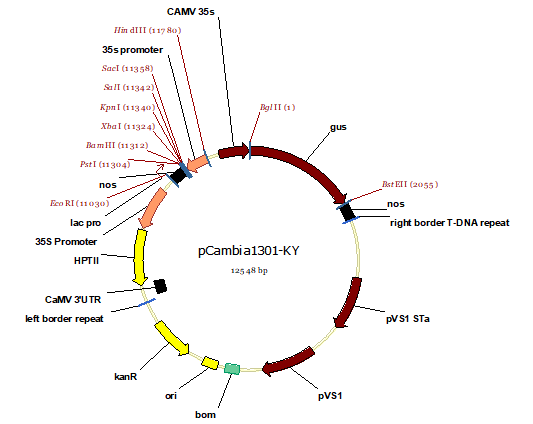 |

Table S3 The information of culture mediums used in this study.

| Name | Main components | Agar (g/L) | Sucrose (g/L) | 6-BA (g/L) | NAA (g/L) | 2,4-D(g/L) | Tim (g/L) | Hyg (g/L) |
| --- | --- | --- | --- | --- | --- | --- | --- | --- |
| MS1-1 / Callus induction medium | MS | 9 | 20 | 2 | 0.02 | 0.1 | 0 | 0 |
| MS1-2 / Regeneration medium | MS | 9 | 20 | 2 | 0.02 | 0 | 0 | 0 |
| MS2-1 | MS | 9 | 20 | 2 | 0.02 | 0.1 | 0.3 | 0 |
| MS2-2 | MS | 9 | 20 | 2 | 0.02 | 0 | 0.3 | 0 |
| MS3-1 | MS | 9 | 20 | 2 | 0.02 | 0.1 | 0.3 | 0.01 |
| MS3-2 | MS | 9 | 20 | 2 | 0.02 | 0 | 0.3 | 0.01 |
| MS4 | MS | 9 | 20 | 0 | 0.5 | 0 | 0.3 | 0.01 |

Note: Tim, Timentin; Hyg, Hygromycin.

Table S4 Effect of genotypes on the shoot regeneration and transformation efficiency of different explants.

|  | | C50DH-1 | C60DH-3 | C65IL-5 | C80DH-3 | C90IL-9 | C100IL-5 | B65IL-5 | B70DH-3 | B80DH-6 | B80IL-2 | B90IL-6 | B100IL-9 |
| --- | --- | --- | --- | --- | --- | --- | --- | --- | --- | --- | --- | --- | --- |
| Regenerat-ion rate | hypocotyl | 65.34±3.28  bc | 66.57±4.01  c | 78.46±2.43  defg | 82.31±2.13  fghi | 85.14±3.12  ij | 62.47±1.94  bc | 84.39±3.08  hi | 78.42±5.32  defg | 66.37±2.07  c | 90.09±3.76  jk | 67.84±4.32  c | 79.41±3.28  defgh |
|  | cotyledon petiole | 67.92±3.46  c | 52.43±4.31  a | 76.41±5.03  de | 73.96±3.46  d | 80.72±4.05  efghi | 60.85±4.38  b | 84.93±2.62  hij | 75.17±3.58  de | 76.85±4.15  def | 82.52±3.04  ghi | 66.96±2.89  c | 83.31±2.54  ghi |
|  | peduncle | 94.63±1.89  kl | 97.14±0.87  l | 95.26±1.42  kl | 94.91±2.06  kl | 98.03±0.53  l | 93.26±1.49  kl | 95.61±2.04  kl | 96.34±0.73  l | 95.99±1.07  l | 97.63±0.59  l | 96.18±1.54  l | 98.34±0.48  l |
| Tansform-ation rate | hypocotyl | 0 | 0 | 0 | 0 | 0.7±0.05  a | 0 | 3.2±0.26  bc | 0 | 0 | 4.8±0.86  c | 0 | 0 |
|  | cotyledon petiole | 0 | 0 | 0 | 0 | 0.3±0.04  a | 0 | 2.1±0.14  ab | 0 | 0 | 3.6±0.38  bc | 0 | 0 |
|  | peduncle | 8.9±0.89  d | 9.7±1.24  dce | 11.8±1.02  fgh | 8.8±0.75  d | 10.6±0.68  dce | 9.4±1.26  dc | 13.4±2.04  h | 9.9±1.51  dce | 9.4±0.93  dc | 12.9±1.69  gh | 10.8±2.06  dce | 11.3±1.26  efg |

Note, different letters (a-l) among the levels of each factor indicate the significant differences between them (P < 0.05).

Table S5 Browning rate of different explants of C90IL-9 and B80IL-2 after different time of *Agrobacterium* infection

| Variety | Explants | Browning rate (%) | | | | | | | |
| --- | --- | --- | --- | --- | --- | --- | --- | --- | --- |
|  |  | 0 min | 5 min | 10 min | 15 min | 20 min | 30 min | 40 min | 60 min |
| C90IL-9 | Peduncles at grade 2/3 | 1.83±0.32ab | / | 2.85±0.46abc | / | 4.23±0.84abcd | 7.36±1.03def | 20.39±2.19mn | 41.28±5.16p |
|  | Hypocotyls | 5.24±0.69bcd | 13.79±1.02hij | 14.34±1.34ijk | 25.31±1.85o | 47.73±3.64q | / | / | / |
|  | Cotyledon petioles | 6.49±0.68cde | 15.47±1.63jkl | 17.69±1.27klm | 26.49±0.95o | 49.43±3.17q | / | / | / |
| B80IL-2 | Peduncles at grade 2/3 | 1.32±0.21a | / | 2.03±0.19ab | / | 4.06±0.53abcd | 6.59±0.69cde | 18.04±1.51lm | 39.0±3.49p |
|  | Hypocotyls | 4.37±0.68abcd | 9.47±1.05efg | 11.32±2.03ghi | 24.27±2.41o | 47.26±3.27q | / | / | / |
|  | Cotyledon petioles | 4.71±0.84abcd | 10.27±1.58fgh | 11.58±1.63ghi | 22.92±2.09no | 41.08±4.04p | / | / | / |

Note, different letters (a-q) among the levels of each factor indicate the significant differences between them (P < 0.05).

**
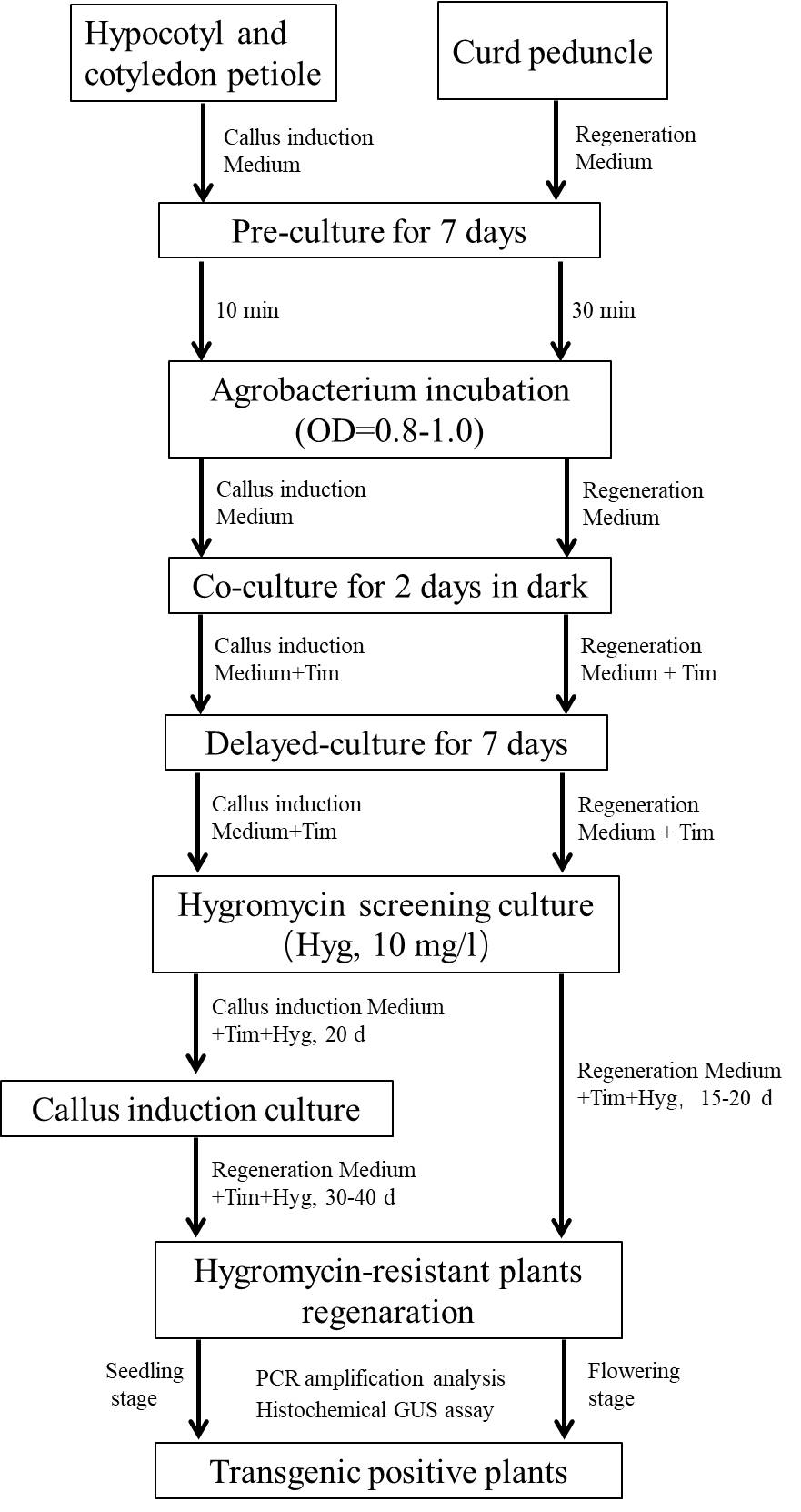
**

**Figure S1.** Overview of the *Agrobacterium*-mediated transformation process in cauliflower and broccoli using different explants. Tim, Timentin.
